# Annular Ellipticity and Sizing Strategy in Transcatheter Aortic Valve Implantation: Independent or Combined Risk Patterns?

**DOI:** 10.1002/ccd.70540

**Published:** 2026-03-01

**Authors:** Emre Polat, Lisa Müller, Lena Schemet, Sarah Friedrich Welz, Mohamed Amer, Evaldas Girdauskas, Tamer Owais

**Affiliations:** ^1^ Department of Cardiothoracic Surgery University Hospital Augsburg Augsburg Germany; ^2^ Department of Mathematical Statistics and Artificial Intelligence in Medicine University of Augsburg Augsburg Germany; ^3^ Center for Advanced Analytics and Predictive Sciences (CAAPS), University of Augsburg Augsburg Germany; ^4^ Department of Cardiac Surgery, Heart Centre Helios Wuppertal University of Witten/Herdecke Wuppertal Germany

**Keywords:** aortic valve, aortic valve stenosis, heart valve, transcatheter aortic valve implantation

## Abstract

**Background:**

Elliptical annular anatomy has been considered a risk factor for adverse outcomes after TAVI, particularly paravalvular leakage (PVL). Prosthesis oversizing is thought to improve sealing, but the interaction between annular shape and sizing strategy remains unclear.

**Aim:**

To evaluate the combined impact of annular shape and sizing strategy on procedural success and short‐term outcomes.

**Methods:**

In this retrospective single‐center study, 509 patients undergoing TAVI between January 2021 and December 2024 were analyzed. Ellipticity index (EI) was defined as the ratio of maximum to minimum annular diameter on preprocedural CT. The sizing index (SI) was calculated as prosthesis diameter relative to mean annular diameter. Primary and secondary endpoints were device success and PVL ≥ mild, PPI, new‐onset LBBB, and early safety.

**Results:**

Mean EI and SI were 1.27 ± 0.10; 15.3% ± 12.4%. Device success was achieved in 89% and was not significantly associated with EI (aOR 0.197, 95% CI 0.013–3.309, *p* = 0.25). Increasing SI was associated with higher device success, although this association did not reach statistical significance (aOR 1.017, *p* = 0.088). Success rates peaked with moderate oversizing (6%–15%; 93%). PVL ≥ mild occurred in 30% with undersizing and 14% with optimal oversizing but showed no association with EI or SI. Heatmaps showed lower device success when high EI (> 1.32) coincided with suboptimal SI (≤ 5% or ≥ 25%).

**Conclusion:**

Annular ellipticity alone did not predict adverse outcomes. While exploratory analysis suggested reduced device success in patients with both high ellipticity and suboptimal sizing, no significant interaction was identified. Moderate oversizing was associated with the most favorable results.

## 1 Introduction

Accurate assessment of aortic annulus geometry is central to the success of transcatheter aortic valve implantation (TAVI). While sizing strategies have traditionally relied on circular assumptions, several studies have shown that the native aortic annulus is frequently elliptical rather than round, particularly in elderly patients with calcific aortic stenosis [1, 2]. This eccentric configuration has raised concerns regarding suboptimal prosthesis apposition, paravalvular leakage (PVL), and the need for post‐dilatation [3].

**Figure 1:**
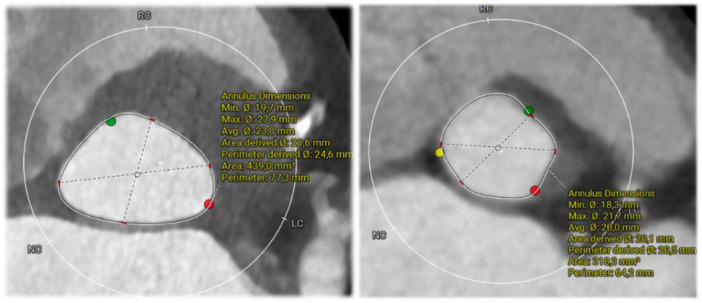
Representative CT cross‐sections of the aortic annulus illustrating an elliptical (*left*) and a near‐circular (*right*) annular geometry. [Color figure can be viewed at wileyonlinelibrary.com]

Multidetector computed tomography (MDCT) enables detailed quantification of annular ellipticity, offering a reproducible imaging‐based assessment prior to TAVI (Figure 1) [4]. Despite theoretical concerns, clinical data on the prognostic impact of annular shape remain conflicting. While some observational studies suggest that elliptical anatomy may predispose to adverse outcomes such as PVL or conduction disturbances [5, 6]. others have failed to demonstrate a significant effect on device success or clinical safety endpoints [7, 8, 9].

Recent in‐vitro and imaging‐based research has provided mechanistic insights, indicating that elliptical annuli may impair optimal prosthesis expansion and sealing, particularly when annular sizing is borderline [10, 11]. These effects may be especially relevant in cases of mismatch between annular geometry and prosthesis design or oversizing strategy. The interplay between annular geometry and device sizing strategy has recently gained attention, with suggestions that suboptimal matching may contribute to adverse procedural outcomes in anatomically borderline cases [12].

Prosthesis oversizing, traditionally used to minimize PVL and ensure secure anchoring, has emerged as a modifiable factor that may interact with anatomical eccentricity. Although moderate oversizing is widely adopted in current practice, it remains unclear whether this strategy can effectively compensate for the risks associated with ellipticity [13]. Notably, oversizing beyond optimal thresholds may also lead to complications such as annular injury, impaired valve expansion, or conduction disturbances [14].

Against this background, the current study aims to systematically evaluate the combined impact of aortic annulus ellipticity and prosthesis sizing index (SI) on procedural and early clinical outcomes in patients undergoing transfemoral TAVI.

## 2 Materials and Methods

### 2.1 Study Design and Patient Population

This retrospective, single‐center observational study screened 607 patients with severe aortic stenosis who underwent transfemoral TAVI at the University Hospital Augsburg between January 2021 and December 2024. All patients had preprocedural MDCT imaging available for annular assessment. Patients were excluded in the presence of bicuspid or unicuspid aortic valve morphology, valve‐in‐valve procedures, non‐transfemoral access routes such as transapical or transaxillary approaches, isolated aortic regurgitation without concomitant stenosis, or inadequate imaging quality precluding annular measurement.

### 2.2 Assessment of Annulus Ellipticity and SI

Preprocedural MSCT datasets were analyzed using dedicated post‐processing software. The ellipticity index (EI) of the aortic annulus quantifies its deviation from a perfect circle by comparing its longest and shortest diameters in the annular plane:

$$\text{EI}\;=D_{\max}/D_{\min}$$

where $D_{\max}$ is the maximum diameter and $D_{\min}\;$the minimum diameter. An EI of 1 indicates a perfectly circular annulus, while values above 1 denote increasing ellipticity. The area‐derived mean diameter was used for all calculations, as this represents the standard method for sizing at our institution. The prosthesis SI was calculated by dividing the nominal prosthesis diameter by the mean annular diameter, as measured on MDCT. The EI and SI were analyzed as continuous variables, and their interaction was explored. While annular dimensions and derived indices were assessed in a standardized manner using CT‐based measurements, the sizing decision was guided by manufacturer‐specific sizing charts. Final valve selection and management of residual gradients were operator‐dependent and based on individual judgment and experience.

Intraprocedural assessment of PVL was routinely performed using root aortography and quantified based on the Sellers classification. In cases of more than mild PVL, balloon postdilatation was generally carried out at the discretion of the implanting physician. Postprocedural PVL evaluation was conducted using transthoracic echocardiography prior to discharge.

### 2.3 Endpoints

The primary endpoints of the study were device success at hospital discharge and the occurrence of paravalvular regurgitation greater or equal than mild, as defined by the Valve Academic Research Consortium‐3 (VARC‐3) criteria. Secondary outcomes included freedom from new permanent pacemaker implantation (PPI) within 30 days, occurrence of new‐onset left bundle branch block (LBBB), and the VARC‐3‐defined early safety endpoint [15].

### 2.4 Statistical Analysis

Categorical data are presented as counts (*n*) and percentages (%), and continuous variables as means ± standard deviation (SD). Group comparisons for categorical variables were performed using the *χ*2 test and Fisher's exact test (when the expected value in any of the cells in the contingency table was < 5). For continuous data, an independent 2‐tailed Student's *t*‐test or Mann–Whitney *U*‐test was used if data were normally or non‐normally distributed, respectively.

To evaluate the impact of annular ellipticity, prosthesis sizing, and their interaction on the endpoints, uni‐ and multivariable logistic regression models were constructed. All models were adjusted for patient age and sex. For selected outcomes, additional covariates were included: EuroScore II was added for analyses related to device success and early safety, asymmetric annular calcium distribution was added for analyses related to PVL; for models addressing new PPI and LBBB, pre‐existing right bundle branch block (RBBB) and implantation depth were considered relevant predictors, and patients with pre‐existing conduction disturbances were excluded. Interaction effects between ellipticity and sizing were analyzed by incorporating a multiplicative interaction term into the regression models. As a sensitivity analysis, we repeated both univariate and multivariate logistic regressions without this interaction term and substituted the eccentricity index for EI in all models.

Finally, to visualize the joint and individual effects of the two variables on the primary outcomes of interest, we visualized ROC curves, spline‐based logistic regression and two‐dimensional heatmaps, depicting predicted outcome probabilities across their observed ranges.

All statistical analyses were performed using R software (version 4.4.2; R Foundation for Statistical Computing, Vienna, Austria). A two‐sided *p* value of < 0.05 was considered statistically significant, and all analyses are considered exploratory, thus not adjusting for multiple testing.

## 3 Results

Out of 607 patients screened, 509 were included in the final analysis following exclusion of those with bicuspid or unicuspid aortic valve morphology (*N* = 30), valve‐in‐valve procedures (*N* = 34), non‐transfemoral access routes (*N* = 29), and isolated aortic regurgitation (*N* = 5). Baseline demographic and clinical variables are summarized in Table 1. Discharge parameters, including postprocedural conductions disturbances, are detailed in Table 2. The mean EI was 1.27 ± 0.10, and the mean SI was 15.3% ± 12.4%.

**Table 1:** Baseline characteristics.

Variables	All patients *N* = 509 *N* (%)	BEV *N* = 348 *N* (%)	SEV *N* = 161 *N* (%)	*p* value
Age [years], mean ± sd	80.4 ± 6.8	79.5 ± 7	82.2 ± 6	0.001
Gender Female	222 (43.6)	116 (33.3)	106 (65.8)	0.001
Body‐Mass‐Index [kg/m²], mean ± sd	27.7 ± 5.6	28 ± 5.9	27.1 ± 4.7	0.096
EuroScore II [%], mean ± sd	4.2 ± 4	4 ± 3.9	4.4 ± 4.2	0.287
Hypertension	452 (88.8)	309 (88.8)	143 (88.8)	0.722
Diabetes mellitus	169 (33.2)	122 (35)	47 (29.33)	0.533
Dyslipidemia	400 (78.6)	274 (78.7)	126 (78.3)	0.789
Prior stroke	64 (12.6)	43 (12.4)	21 (13)	0.065
COPD	64 (12.6)	48 (13.8)	16 (9.9)	0.450
Immobility	75 (14.7)	54 (15.5)	21 (13)	0.356
Prior myocardial infarction	118 (23.2)	78 (22.4)	40 (24.8)	0.623
Chronic kidney disease	131 (25.7)	92 (26.4)	39 (24.2)	0.673
Dialysis	13 (2.6)	12 (3.4)	1 (0.6)	0.115
NYHA IV	30 (5.9)	20 (5.7)	10 (6.2)	0.397
Atrial fibrillation	142 (27.9)	98 (28.2)	44 (27.3)	0.935
Prior permanent pacemaker	26 (5.1)	17 (4.9)	9 (5.6)	0.812
Intraventricular conduction disturbances (LAHB, LBBB, RBBB)	135 (26.5)	88 (25.4)	47 (29.2)	0.473

**Table 2:** Procedural characteristics.

Variables	All patients *N* = 509 *N* (%)	BEV *N* = 348 *N* (%)	SEV *N* = 161 *N* (%)	*p* value
Analgesia	460 (90.4)	308 (88.5)	152 (94.4)	0.053
Prosthesis size [mm], mean ± sd	26 ± 2.3	25.9 ± 2.2	26 ± 2.5	0.587
Prothesis type				
balloon‐expandable	348 (68.4)	348 (68.4)	0 (0)	0.001
self‐expanding	161 (31.6)	0 (0)	161 (100)	
Edwards Sapien Ultra 3	337 (66.2)	337 (96.8)	0 (0)	0.001
Boston Acurate Neo 2/Acurate Prime	114 (22.4)	0 (0)	114 (70.8)	0.001
Medtronic Evolut Pro/Evolut FX+	47 (9.2)	0 (0)	47 (29.2)	0.001
Myval Octacor	11 (2.2)	11 (3.2)	0 (0)	0.001
Procedure time [min], mean ± sd	40.3 ± 23.4	38.1 ± 18.3	45.1 ± 31.2	0.001
Fluoroscopy time [min], mean ± sd	8.9 ± 4.9	8.5 ± 4.7	9.7 ± 5.2	0.012
Dose area product [Gycm^2^], mean ± sd	2206.2 ± 7037.1	2317.5 ± 8173.5	1965.7 ± 3500.7	0.600
Contrast dosage [mL], mean ± sd	72.1 ± 36.1	65.1 ± 31.7	87.3 ± 40.2	0.001
Predilatation	359 (70.5)	202 (58)	157 (97.5)	0.984

### 3.1 Primary Endpoint Analyses

Device success was achieved in 89% (454/509) of the patients, with consistently high rates and did not significantly differ across varying degrees of annular ellipticity (range 88%–91%, *p* = 0.78). Likewise, the EI, when modeled as a continuous variable, was not independently associated with device success (adjusted OR: 0.197, 95% CI: 0.013–3.309, *p* = 0.25) (Table 3). Similarly, the SI did not reach statistical significance (adjusted OR: 1.017, 95% CI: 0.998–1.039, *p* = 0.088), with descriptively higher success rates of 93% observed in the subgroup with moderate oversizing (6%−15%), compared to those with minimal oversizing (≤ 5%) or aggressive oversizing (³ 20%) (85% and 79%, respectively) (Table 4). The interaction term between EI and SI was also not statistically significant (adjusted OR: 1.063, 95% CI: 0.869–1.312, *p* = 0.563).

**Table 3:** Uni‐ and multivariable logistic regression models evaluating the association between Ellipticity‐Index and defined endpoints.

	Univariable			Multivariable		
	OR	95% CI	*p* value	OR	95% CI	*p* value
Device success	0.208	0.014‐3.483	0.266	0.197	0.013‐3.309	0.250
New LBBB	0.573	0.063‐4.875	0.614	0.734	0.081‐6.325	0.781
Early safety	0.270	0.036‐2.029	0.2	0.270	0.036‐2.037	0.201
Paravalvular leakage	0.645	0.059‐6.537	0.714	0.708	0.062‐7.478	0.777
Freedom from new PPI	0.298	0.026‐3.661	0.337	0.359	0.030‐4.552	0.422

Abbreviations: CI, confidence interval; LBBB, left bundle branch block; OR, odds ratio; PPI, permanent pacemaker implantation.

**Table 4:** Uni‐ and multivariable logistic regression models evaluating the association between Sizing–Index and defined endpoints.

	Univariable			Multivariable		
	OR	95% CI	*p* value	OR	95% CI	*p* value
Device success	1.016	0.998–1.037	0.104	1.017	0.998–1.039	0.088
New LBBB	1.005	0.992–1.017	0.423	1.005	0.992–1.018	0.441
Early safety	0.996	0.984–1.008	0.487	0.996	0.984–1.008	0.486
Paravalvular leakage	0.998	0.983–1.011	0.754	0.998	0.983–1.012	0.766
Freedom from new PPI	0.995	0.980–1.010	0.474	0.995	0.980–1.010	0.477

Abbreviations: CI, confidence interval; LBBB, left bundle branch block; OR, odds ratio; PPI, permanent pacemaker implantation.

The occurrence of PVL ≥ mild was likewise not associated with annular ellipticity, with the EI showing no significant effect in multivariable analysis (adjusted OR 0.708, 95% CI 0.062–7.478, *p* =  0.777). Similarly, the SI showed no independent association with PVL (adjusted OR 0.998, 95% CI 0.983–1.012, *p* =  0.766), and no significant interaction between the two parameters was observed (adjusted OR for EI × SI interaction 1.066, 95% CI 0.910–1.246, *p* =  0.422). Descriptively, PVL ≥ mild occurred in 30% of patients with undersizing (≤–5%), compared to 14% with moderate oversizing (6%–15%), and ranged from 13% to 24% across ellipticity subgroups without a discernible pattern (Supporting Information S1: Figure 1).

### 3.2 Secondary Endpoint Analyses

None of the secondary endpoints—including freedom from new PPI, new‐onset LBBB, and the VARC‐3‐defined early safety endpoint—showed a significant association with either EI or SI. Freedom from new PPI within 30 days was not significantly influenced by the EI (adjusted OR: 0.785, 95% CI: 0.092–6.376, *p* = 0.822) or the SI (adjusted OR: 1.009, 95% CI: 0.996–1.021, *p* = 0.164). Similarly, early safety events were not significantly influenced by annular shape (adjusted OR: 0.270, 95% CI: 0.036–2.037, *p* = 0.201) and SI (adjusted OR: 0.996, 95% CI: 0.984–1.008, *p* = 0.486). New‐onset LBBB also occurred independently of both parameters (EI: adjusted OR 0.734, 95% CI: 0.081–6.325, *p* = 0.781; SI: adjusted OR 1.005, 95% CI: 0.992–1.018, *p* = 0.441).

### 3.3 Interaction and Explorative Analysis

**Figure 2:**
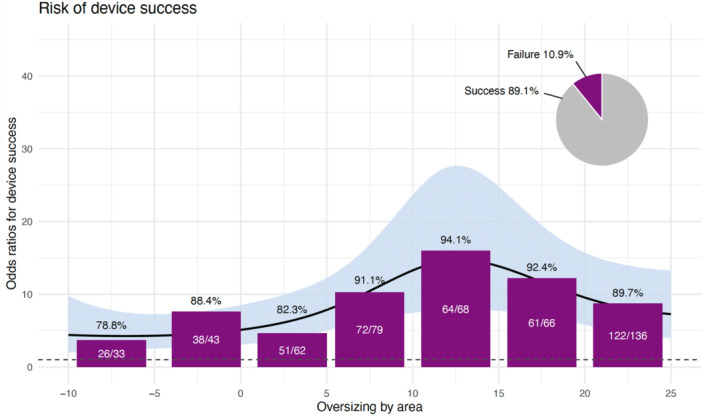
Relationship between SI and device success: device success: the black spline curve with 95% confidence interval (blue shaded area) depicts the modeled odds of success. Peak success was observed with moderate oversizing (6%−15%). [Color figure can be viewed at wileyonlinelibrary.com]

Figure 2 demonstrates that device success rates were highest with moderate oversizing (SI 6%–15%), irrespective of annular ellipticity, and lowest in cases of pronounced undersizing (SI ≤ 5%) or excessive oversizing (SI ≥ 25%).

**Figure 3:**
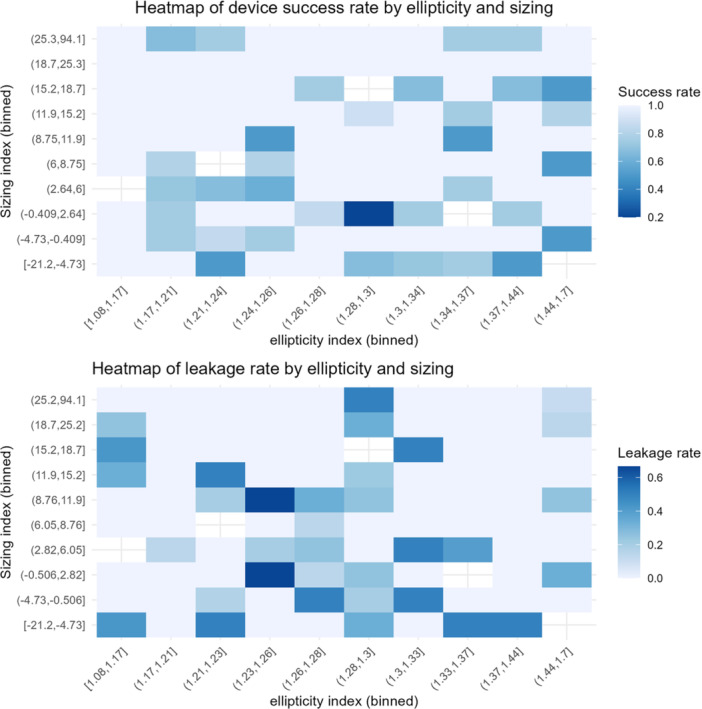
Two‐dimensional heatmaps illustrating the interaction between annular ellipticity and prosthesis sizing index regarding device success (top) and paravalvular leakage (bottom). [Color figure can be viewed at wileyonlinelibrary.com]

Heatmap analysis (see Figure 3 **)** further revealed a marked reduction in device success (< 80%) in the subgroup with EI > 1.32 combined with suboptimal sizing (SI ≤ 5% or ≥ 25%). While these patterns may suggest a potential interplay between aortic annular shape and sizing strategy, they did not translate into a statistically significant interaction in our multivariable analysis and should therefore be interpreted as exploratory. In contrast, no distinct ellipticity threshold could be identified that reliably predicted procedural failure or PVL, underscoring the importance of integrated sizing strategies over absolute annular shape metrics.

The spline model (see Supporting Information S1: Figure 2) complements this pattern by showing only minimal variation in the predicted probability of device success across the EI spectrum, reinforcing the notion that annular shape alone does not exert a clinically meaningful impact on procedural outcomes.

## 4 Discussion

This study investigated the combined effects of aortic annular ellipticity and prosthesis sizing on outcomes following transfemoral TAVI. Our findings demonstrate that while neither ellipticity nor SI alone was significantly associated with device success or PVL, exploratory analysis revealed patterns suggesting that the combination of pronounced ellipticity with undersizing or excessive oversizing may be associated with reduced procedural success, although no significant interaction was identified. These findings imply that although sizing extremes and annular shape might influence sealing behavior, neither factor independently predicted PVL risk in this cohort. The descriptive reduction in device success observed in anatomies with both high EI and suboptimal SI underscores the potential importance of geometry‐adapted sizing strategies in TAVI.

Our results contrast with those of Tomii et al., who found no significant association between annular ellipticity and composite early device failure using contemporary devices (SAPIEN 3 and Evolut R). Their findings support our multivariable analysis, where annular shape alone was not predictive of device failure. However, unlike their study, which did not assess potential interactions with prosthesis sizing, our exploratory heatmap analysis indicated that procedural outcomes may be less favorable when annular eccentricity coincides with suboptimal sizing, despite the absence of a significant interaction [4].

The biomechanical study by Kuetting et al. demonstrated that increasing ovality of the annulus was associated with higher transvalvular gradients and increased regurgitation in vitro when self‐expanding devices were tested. While our clinical results did not show a direct correlation between ellipticity and PVL, we did observe descriptively higher PVL rates in undersized prostheses, supporting the mechanical plausibility of these laboratory findings [16].

Mas‐Peiro et al. identified excessive oversizing and deep valve implantation as key predictors of new PPI after balloon‐expandable TAVI. While our analysis did not confirm an independent effect of SI on PPI risk, this discrepancy may stem from our broader device spectrum or different baseline implantation practices [17]. Similarly, Elnwagy et al. showed that oversizing > 15% increased PPI rates among patients with small annuli, echoing our descriptive trend toward more conduction disturbances at higher sizing indices [18].

In terms of PVL, Kim et al. demonstrated that moderate oversizing (8%–20%) significantly reduced the risk of PVL ≥ mild without increasing annular injury, whereas excessive oversizing yielded diminishing benefits [19]. These results align closely with our clinical data showing the most favorable outcomes in the 6%–15% SI range.

Wen et al. used computational simulations to show that elliptical annuli, especially in combination with misaligned prostheses, exhibited increased stress on aortic walls and a higher risk of adverse biomechanical outcomes [20]. While our clinical outcomes were not directly linked to such modeling endpoints, the observed patterns in patients with EI > 1.32 and suboptimal SI are consistent with their suggestion that elliptical anatomy may confer greater procedural risk when geometric mismatch exists.

Taken together, our findings contribute to a growing body of evidence suggesting that isolated geometric indices may be insufficient for guiding optimal device selection. Instead, integrating annular morphology and sizing strategy appears essential to achieve consistent procedural success. This has practical implications for preprocedural planning, emphasizing the need for individualized sizing in patients with non‐circular annuli.

## 5 Limitations

This study has several limitations. First, its retrospective, single‐center design may introduce selection bias and limit generalizability to broader patient populations or different procedural settings. Patients with bicuspid aortic valves were excluded to maintain a homogeneous study population; therefore, the findings may not be generalizable to bicuspid anatomies. Second, while we adjusted for key clinical and anatomical confounders in multivariable models, residual confounding cannot be entirely excluded. Third, the definition of ellipticity and sizing was based on MDCT‐derived anatomical measurements, which, although standardized, may vary depending on measurement technique and image quality. Formal assessment of inter‐observer variability was not performed, which may affect the reproducibility of measurements. Furthermore, SI was derived using nominal prosthesis diameter, which does not account for deployment‐related variability such as incomplete expansion or eccentric positioning. Moreover, the heatmap analysis was exploratory in nature and based on model‐derived estimates rather than patient‐level subgroup testing. Due to the limited number of events in critical strata, these visual patterns should be interpreted with caution and regarded as hypothesis‐generating. Finally, long‐term clinical outcomes were not assessed in this analysis, and therefore the prognostic implications of ellipticity and oversizing beyond the early postprocedural phase remain uncertain.

## 6 Conclusion

In this large, contemporary TAVI cohort, annular ellipticity alone was not an independent predictor of device success or PVL. Exploratory analysis suggested that unfavorable outcomes may occur in anatomies with high ellipticity and suboptimal prosthesis sizing. Moderate oversizing between 6% and 15% was associated with the highest procedural success, even in elliptical anatomies. These findings underscore the importance of geometry‐adaptive sizing strategies and highlight the need for individualized preprocedural planning to optimize outcomes in TAVI patients.

## 7 Clinical Perspectives

Annular ellipticity and prosthesis are considered key anatomical and procedural factors potentially influencing TAVI outcomes. However, their individual and combined clinical impact remains debated. This study demonstrates that neither annular ellipticity nor prosthesis SI independently predicts device success or PVL after TAVI, although descriptive analysis revealed most favorable outcomes when moderate oversizing was applied—even in patients with markedly elliptical anatomies. Future studies should explore whether individualized sizing protocols—incorporating annular shape—can further improve procedural safety and durability in TAVI.

## Funding

The authors received no specific funding for this work.

## Disclosure

The authors have nothing to report.

## Ethics Statement

The research reported in this paper adhered to STROBE guidelines. Approved by the local Ethics Committee on June 21st, 2024 (ID 24‐0458).

## Consent

The authors confirm that patient consent is not applicable to this article. This is a retrospective study using de‐identified data, therefore the IRB did not require consent from the patient.

## Conflicts of Interest

The authors declare no conflicts of interest.

## Supporting information

Supplementary Material.docx.

## Data Availability

The author takes responsibility for all aspects of the reliability and freedom from bias of the data presented and their discussed interpretation.
