# Supplementary material for: Annular Ellipticity and Sizing Strategy in Transcatheter Aortic Valve Implantation: Independent or Combined Risk Patterns?
Source: Catheter Cardiovasc Interv. 2026 Mar 1;107(6):1774–81. doi: 10.1002/ccd.70540 (PMC13176684; doi:10.1002/ccd.70540)
Supplement: Supplementary file 1 — Supplementary Material.docx. [file CCD-107-1774-s001.docx]

**Supplementary Material**

**Figure S1.**
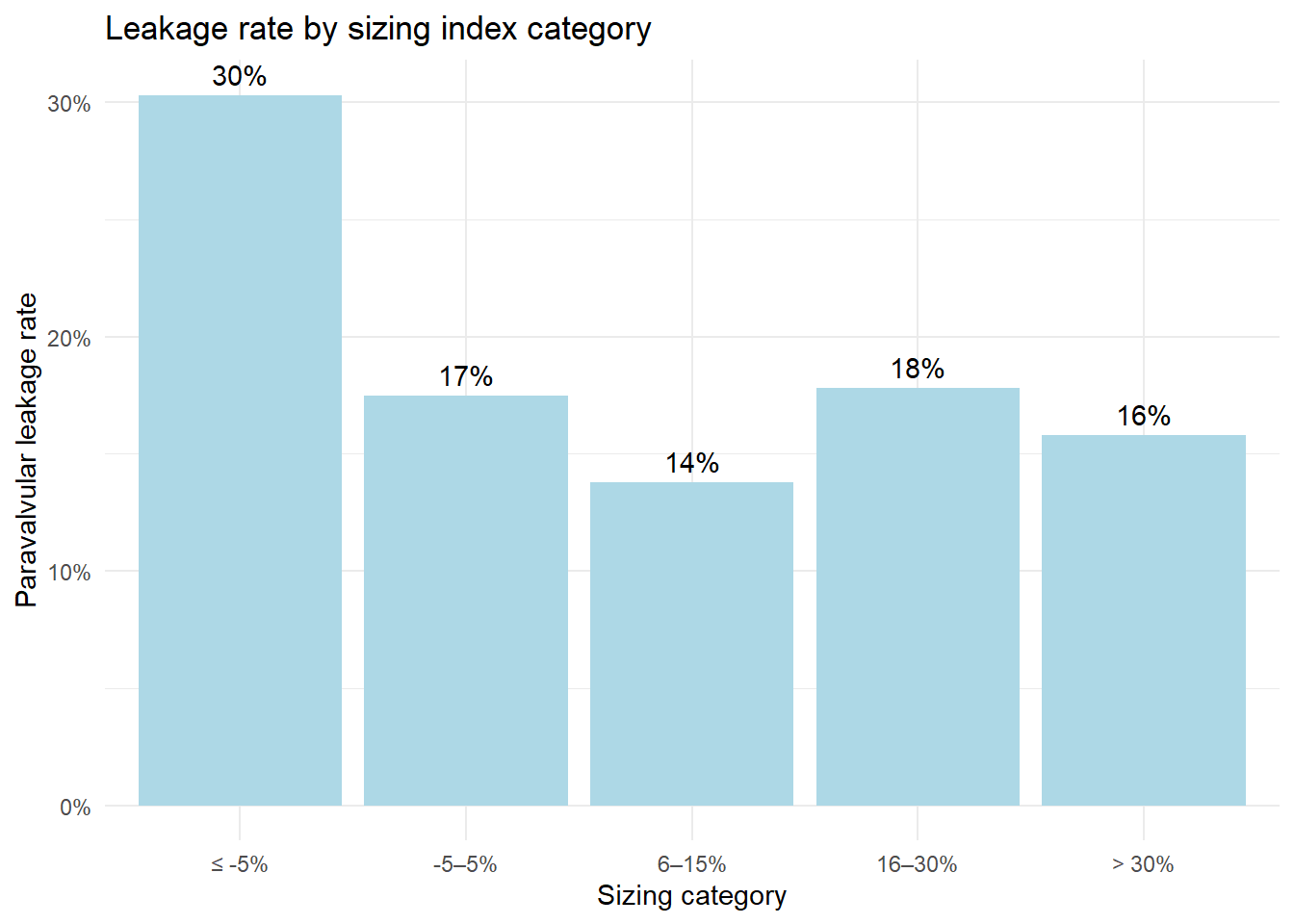


**Figure S2.**


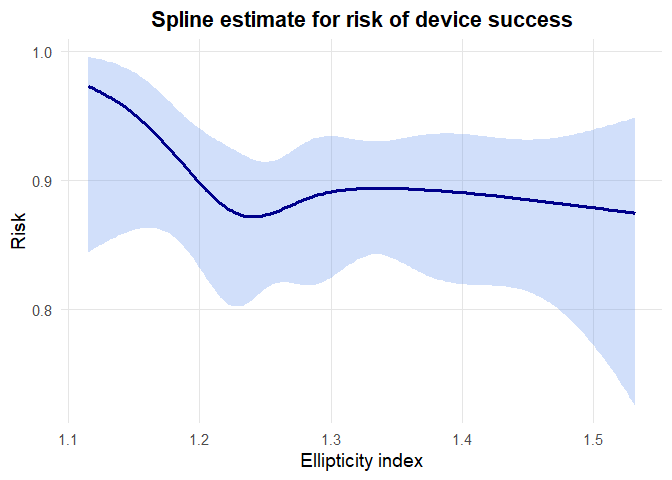


**Table S1.**

| **Combined Endpoints** | **Variables** | **All patients**  **N = 509**  N (%) |
| --- | --- | --- |
| Device success  (at discharge) | *Technical success* | 490 (96.3) |
|  | *Freedom from mortality* | 507 (99.6) |
|  | *Freedom from surgery or intervention related to the device or to a major vascular or access-related or cardiac structural complication* | 478 (93.9) |
|  | *Intended performance of the valve (mean gradient <20mmHg, peak velocity 3m/s, Doppler velocity index ≥ 0.25, and less than moderate aortic regurgitation)* | 485 (95.3) |
| Early Safety  (at 30 days) | *Freedom from all-cause mortality* | 502 (98.6) |
|  | *Freedom from all stroke* | 488 (96.3) |
|  | *Freedom from VARC type 2-4 bleeding* | 478 (94.3) |
|  | *Freedom from major vascular, access-related, or cardiac structural complication* | 481 (94.5) |
|  | *Freedom from acute kidney injury stage 3 or 4* | 485 (98.4) |
|  | *Freedom from moderate or severe aortic regurgitation* | 497 (98.6) |
|  | *Freedom from new permanent pacemaker due to procedure-related conduction abnormalities* | 392 (85.2) |
|  | *Freedom from surgery or intervention related to the device* | 503 (99.2) |
